# Intrapartum anti-disseminated intravascular coagulation therapy leading to successful vaginal delivery following intrauterine fetal death caused by placental abruption: a case report

**DOI:** 10.1186/1752-1947-8-461

**Published:** 2014-12-23

**Authors:** Michiko Honda, Shigetaka Matsunaga, Sumiko Era, Yasushi Takai, Kazunori Baba, Hiroyuki Seki

**Affiliations:** Center of Maternal, Fetal and Neonatal Medicine, Saitama Medical Center, Saitama Medical University, 1981 Kamoda, Kawagoe, Saitama, 350-8550 Japan

**Keywords:** DIC, Intrauterine fetal death, Placental abruption, Vaginal delivery

## Abstract

**Introduction:**

Disseminated intravascular coagulation due to placental abruption with intrauterine fetal death is not uncommon. It can result in increased maternal mortality rates and the need for hysterectomy or greater transfusion volumes if the delivery is not completed within six to eight hours. However, consensus is lacking regarding the delivery approach for cases in which delivery is prolonged.

**Case presentation:**

A 37-year-old Japanese woman was transported to our tertiary center two and a half hours after the onset of labor because of a diagnosis of placental abruption with intrauterine fetal death at 40 weeks and three days’ gestation. On arrival, although severe hypofibrinogenemia was observed, there was no external hemorrhage. Because her cervical canal dilation was good (Bishop score, 7), labor was induced using oxytocin. Anti-disseminated intravascular coagulation therapy was simultaneously started via transfusion. After her hypofibrinogenemia resolved, delivery progressed rapidly, and the fetus was delivered approximately 10 hours after the onset. To reduce postpartum hemorrhage, 6g of fibrinogen concentrate and tranexamic acid, an antifibrinolytic agent, were administered immediately before extraction of the dead fetus and placenta. Although the amount of intrapartum hemorrhage was 1824g, there was no abnormal bleeding after delivery, and our patient was discharged three days later.

**Conclusion:**

In cases of placental abruption complicated with disseminated intravascular coagulation, intrapartum administration of coagulation factors can simultaneously promote effective labor and correct hypofibrinogenemia, enabling minimally invasive vaginal delivery.

## Introduction

**Figure 1:**
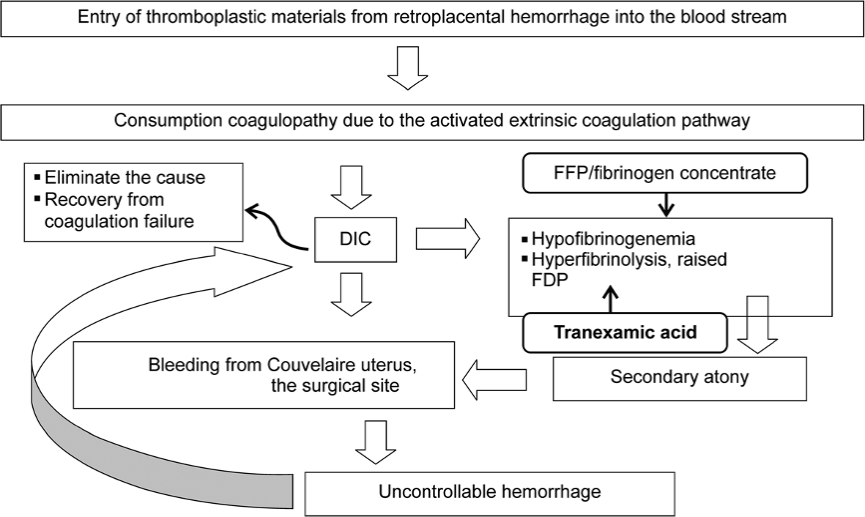
**Therapeutic strategy for placental abruption and uterine atony.** FDP, fibrinogen degradation products; FFP, fresh frozen plasma; PC, platelet concentrate; RCC, red cell concentrate.

Placental abruption occurs when the placenta separates from the uterine wall before delivery and is a major cause of severe maternal complications. Placental abruption occurs in approximately 1% of pregnancies [1], and the incidence of intrauterine fetal death (IUFD) or neonatal death is reported at 20% to 40% [2]. Disseminated intravascular coagulation (DIC) occurs in 10% of all placental abruption cases; the incidence of DIC due to placental abruption is higher in cases with fetal death. Hypofibrinogenemia, elevated levels of fibrinogen degradation products (FDP), and decreased clotting factor activity occur in 30% of cases of placental abruption with IUFD [3]. In cases of DIC due to placental abruption, the tissue factor thromboplastin flows from a retroplacental hematoma into the maternal circulation, resulting in consumption coagulopathy via the activation of an extrinsic coagulation cascade [4]. In addition, hypofibrinogenemia and increased levels of FDP result in secondary uterine atony [5] that can hinder effective labor, increase hemorrhaging from the abruption surface or wound, and aggravate DIC. Anti-DIC therapy requires elimination of the causative disease and coagulation factor supplementation, and a delay in therapy contributes to maternal death (Figure 1).

Minimally invasive vaginal delivery with appropriate anti-DIC therapy is considered first-line therapy, except when external hemorrhage cannot be controlled even with transfusion or when vaginal delivery is difficult because of obstetric complications [6]. Hemorrhage from an abdominal wall wound or uterine wound may actually foster DIC in a Cesarean section, particularly with coagulopathy. In vaginal delivery, massive hemorrhage from the abruption surface can be avoided by appropriate postpartum treatment, such as oxytocin administration and uterine compression [7].

However, there is still insufficient evidence supporting the benefits of vaginal delivery, and consensus regarding the appropriate mode of delivery has not been reached [8]. In the presence of DIC, vaginal delivery presents two clinical problems. First, delivery should be completed within six to eight hours after the onset of placental abruption. Otherwise, DIC may worsen, massive hemorrhage may occur, and the risk of shock and organ damage may increase [9]. Second, with IUFD that is a result of placental abruption, effective labor occasionally does not occur [7]. If delivery is prolonged, DIC may become more severe, and a larger volume of blood transfusion or a hysterectomy may be required [10].

Here, we report the case of a patient in whom vaginal delivery was selected after placental abruption with IUFD. With the simultaneous correction of hypofibrinogenemia, effective labor was achieved, and DIC was quickly controlled after vaginal delivery approximately 10 hours after onset.

## Case presentation

A 37-year-old Japanese woman with a history of five pregnancies and three full-term vaginal deliveries with no co-morbidities or diseases was examined by her obstetrician because of amenorrhea lasting 14 months after her previous delivery. She did not smoke and had no history of abruption or thromboembolism. Her gestational age was estimated at 28 weeks and two days based on the biparietal diameter of her fetus. No placental or fetal abnormalities were detected on ultrasonography. Hypertensive disease was not observed prior to or during the pregnancy.

At 40 weeks and three days of gestation, she was examined by her obstetrician at 3:20 AM owing to lower abdominal pain that had started at 2:00 AM. Ultrasonography revealed a retroplacental hematoma and no fetal heartbeat. With a diagnosis of placental abruption with IUFD, emergency care at our tertiary center was requested, and our patient arrived at 4:30 AM.

**Figure 2:**
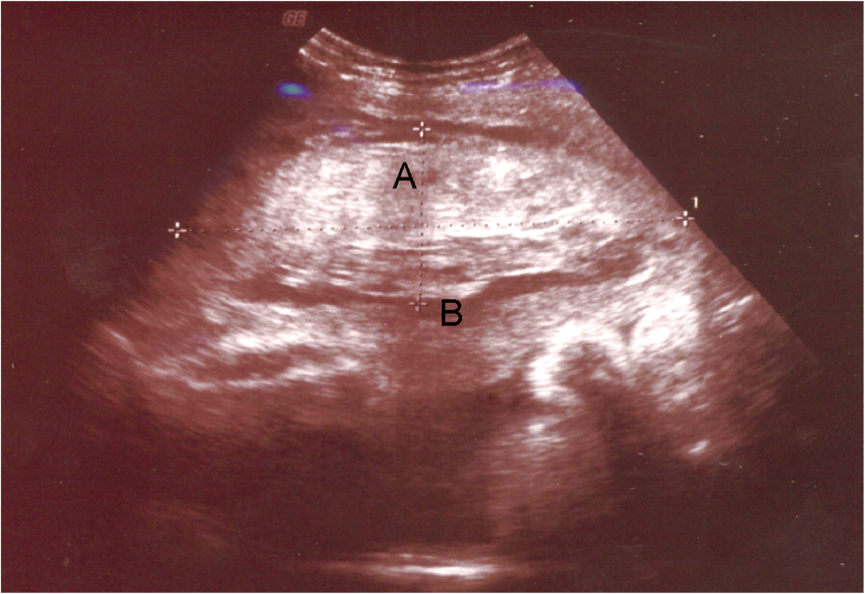
**Ultrasonography findings with placental abruption. (A)** Retroplacental hematoma. **(B)** Placenta.

A physical examination of our patient on admission revealed lucid consciousness, a body temperature of 36.5°C, blood pressure of 116/85mmHg, and pulse rate of 75 beats per minute. She had mild abdominal pain and her uterus was painfully tender. On abdominal ultrasonography, the fetus had a cephalic presentation, and no heartbeat was observed. Her placenta was attached to the anterior wall, and a 9.5×4.6cm internal retroplacental hematoma was observed (Figure 2). Echo-free space suggesting uterine rupture or other intra-abdominal hemorrhage was not present. A pelvic examination revealed no vaginal hemorrhage and a cervical os dilation of 3cm. Effacement was 60%, and station was -2 (Bishop score: 7). Uterine contractions were three minutes apart according to cardiotocography.

Her blood test findings on admission at 4:30 AM were as follows: white blood cell count 11,200/μL, red blood cell count 207×10^4^/μL, hemoglobin 6.5g/dL, hematocrit 19.6%, platelets 100×10^3^/μL, activated partial thromboplastin time 56.5 s, international normalized ratio of prothrombin time 1.85, fibrinogen <70mg/dL, FDP 52.2μg/mL, D-dimer 32μg/mL, C-reactive protein 0.0mg/dL, aspartate aminotransferase 18IU/L, alanine aminotransferase 10IU/L, lactate dehydrogenase 269IU/L, creatine kinase 133U/L, total bilirubin 0.9mg/dL, total protein 5.1g/dL, albumin 2.7g/dL, blood urea nitrogen 11mg/dL, creatine 0.64mg/dL, sodium 137mEq/L, potassium 3.7Eq/L, and chlorine 111mEq/L. Her International Society on Thrombosis and Haemostasis DIC score was 5 (overt DIC).

**Figure 3:**
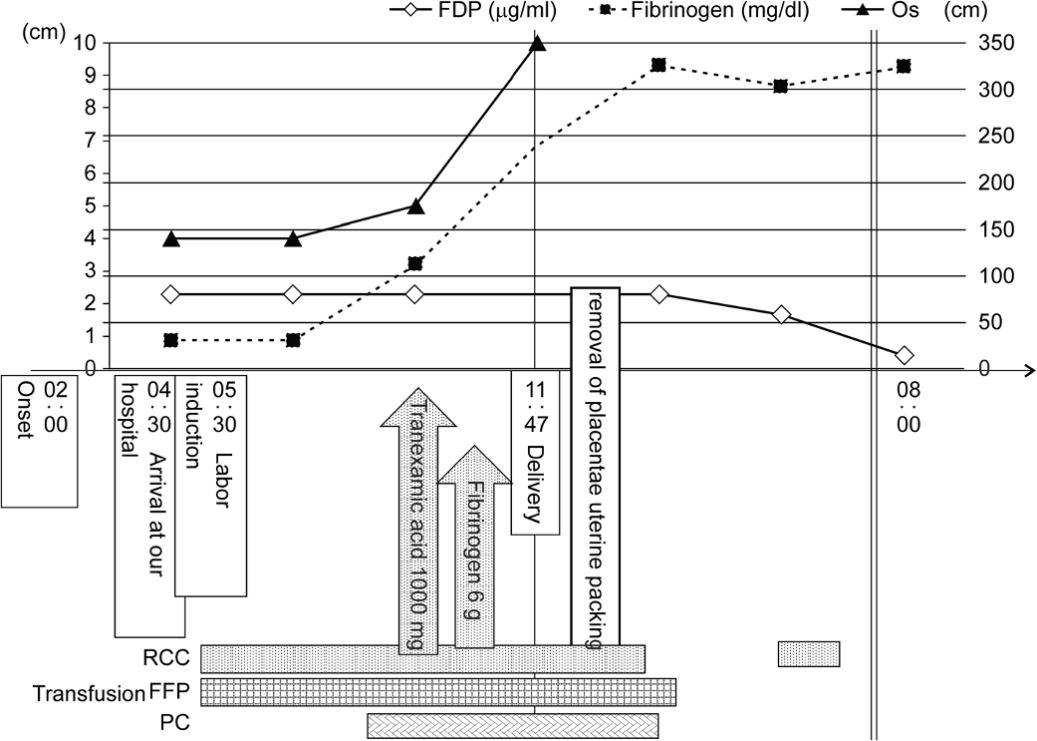
**Clinical course and administration of coagulation factors in placental abruption.** DIC, disseminated intravascular coagulation; FDP, fibrinogen degradation products; FFP, fresh frozen plasma.

Vaginal delivery was selected because her vital signs were stable, no signs of organ failure were detected with the laboratory tests, and her cervical canal dilation was good. Labor was induced by administering incremental doses of oxytocin at 2mIU/mL every 30 minutes from 5:30 AM. Transfusion of red cell concentrate (RCC) and fresh-frozen plasma (FFP) was also started. Her uterine contractions increased from 6:00 AM, but effective labor was not achieved. At 9:00 AM, her blood fibrinogen level was <70mg/dL with no improvement in DIC although a total of eight units of RCC and 24 units of FFP had been transfused. At this time, amniotomy was performed in anticipation of labor progression, but her cervical os was still 5cm with no remarkable progression at 10:00 AM. At 10:30 AM, her blood fibrinogen level increased to 112mg/dL, and labor progressed rapidly. To reduce the amount of hemorrhaging, 1000mg of the antifibrinolytic agent tranexamic acid and 6g of fibrinogen concentrate were administered immediately before delivery. At 11:47 AM, a dead fetus and placenta, together with 900g of blood and gelosis, were extracted. Oxytocin was administered, and bimanual compression of her uterus was performed. Because slight uterine atony was noted, her uterus was packed with gauze to prevent additional bleeding. No birth canal injury was seen. Her blood fibrinogen level at 2:00 PM was 326mg/dL, and DIC was dissolved quickly with no subsequent abnormal hemorrhaging. The time required for labor was nine hours and 47 minutes from its initial onset, the amount of intrapartum hemorrhage was 1824g, and the total transfused volume was 12 units of RCC, 30 units of FFP, 20 units of platelet concentrate, 6g of fibrinogen concentrate, and 3000 units of human antithrombin concentrate. The weight of the placenta was 420g, and approximately 50% of the placenta appeared to be abrupted. The fetus was a male weighing 3024g with no congenital defect. After the gauze was removed the next morning, day three, our patient was discharged (Figure 3).

## Discussion

Consensus regarding the mode of delivery in cases of placental abruption associated with IUFD has not yet been reached [9]. In Japan, obstetricians are more likely to choose a Cesarean section and eliminate the cause of DIC, compared with those in western countries, because blood coagulation factor preparations such as fibrinogen concentrate and cryoprecipitates are not covered by the Japanese national health insurance system. However, safe and successful vaginal deliveries have been increasingly reported in cases of placental abruption with IUFD in Japan. In 506 cases of placental abruption with IUFD extracted from the Japan Society of Obstetrics and Gynecology Perinatal Registration Database between 2002 and 2008, Cesarean sections accounted for 87.5% and 66.6% of the deliveries in 2002 and 2008, respectively [11], suggesting an increase in the number of obstetricians opting for vaginal delivery.

Delivery should be completed within six to eight hours after the onset of placental abruption to avoid worsening of DIC and organ damage [9]. Effective labor is required for prompt vaginal delivery, but there are occasional cases of placental abruption with IUFD in which labor is not initiated [7]. Imanaka *et al.* suggested that immediate amniotomy is the key to successful vaginal delivery [12]. In our case, the delayed amniotomy may have prolonged the labor. However, we also noted that effective labor was observed at the same time as the improvement in our patient’s blood fibrinogen level. Therefore, the coagulation factors in the FFP may have promoted effective uterine contractions, similar to uterine atony caused by DIC.

Furthermore, in our case, hypofibrinogenemia was quickly corrected by fibrinogen concentrate immediately before extraction of the dead fetus and placenta. Because of the increase in the levels of FDP, which is considered a cause of uterine atony, the antifibrinolytic agent tranexamic acid was administered after extraction [13, 14], and sufficient uterine compression was performed, preventing massive hemorrhage after delivery. With the intrapartum and postpartum replacement of coagulation factors, a safe vaginal delivery may be accomplished even if the labor is prolonged slightly more than the recommended six to eight hours. However, our patient did not experience shock or organ failure such as renal failure. If these occur, labor induction should be ceased and a Cesarean section should be performed immediately because these conditions are improved only by delivery. Because the effect of delayed delivery on the outcomes is not fully known, further investigation is needed in additional cases.

## Conclusion

Intrapartum anti-DIC therapy may help the onset of effective labor with minimal hemorrhage in cases of DIC-complicated placental abruption.

## Consent

Written informed consent was obtained from the patient for publication of this case report and accompanying images. A copy of the written consent is available for review by the Editor-in-Chief of this journal.
